# UV-B Resistance in *Artemia*: A Comparative Analysis Across Altitudinal Gradients, Development Stages, and Reproductive Modes

**DOI:** 10.3390/biology14121763

**Published:** 2025-12-10

**Authors:** Jiawei Xu, Cheng Ma, Bingheng Chen, Yunhao Guo, Malik Qammar, Yingguo Gan, Xiaoqi Yu, Zhichao Wang

**Affiliations:** State Key Laboratory Incubation Base for Conservation and Utilization of Bio-Resource in Tarim Basin, College of Life Science and Technology, Tarim University, Alar 843300, China; 15165235877@163.com (J.X.);

**Keywords:** *Artemia*, UV-B tolerance, reproductive modes, altitude, development stages

## Abstract

The *Artemia* exhibit broad environmental adaptability and distinct reproductive modes, making them ideal for studying stress tolerance. Solar UV-B radiation poses a threat to aquatic organisms; however, for *Artemia*—a key aquatic organism characterized by a wide altitudinal distribution range and unique reproductive modes—it remains unclear how their UV-B resistance varies with altitudinal gradients, developmental stages, and reproductive modes. Here, we evaluated six *Artemia* species/lineages (three bisexual species, three parthenogenetic lineages) from 113 to 4700 m altitude across three developmental stages (embryo, nauplius, adult) under gradient UV-B doses. Key results: (1) UV-B resistance showed a dose-dependent positive correlation with altitude, especially under high-dose UV-B (6.4 kJ·m^−2^, R^2^ = 0.72, *p* < 0.01); (2) Adults had the highest resistance, while nauplii were the most vulnerable; (3) Bisexual species had 41–57% higher adult survival rates than parthenogenetic lineages at similar altitudes, but parthenogenetic lineages showed higher embryo relative hatching rates. These findings clarify the adaptive traits of *Artemia* in response to UV-B stress.

## Simple Summary

*Artemia* is an ideal model for stress tolerance studies due to its broad environmental adaptability and distinct reproductive modes. However, how its UV-B resistance varies with altitudinal gradients, developmental stages, and reproductive modes remains unclear. By using six *Artemia* species/lineages from 113–4700 m across embryo, nauplius, and adult stages under gradient UV-B doses, this study reveals the following: UV-B resistance positively correlates with altitude, especially under high-dose UV-B; adults are the most resistant; nauplii are the most vulnerable; bisexual adults survive better than parthenogenetic lineages; and parthenogenetic embryos show higher relative hatching rates. These discoveries clarify *Artemia*’s adaptive traits in response to UV-B stress and provide important insights for research on biological adaptation to extreme environments.

## 1. Introduction

UV-B radiation (280–315 nm) is a ubiquitous environmental factor influencing the evolution and ecology of organisms worldwide, with levels rising in tandem with solar altitude angle [1,2,3,4]. The higher the altitude, the larger the solar altitude angle, and consequently, the greater the UV-B radiation. The thin atmospheres and historical ozone depletion of high-altitude regions result in persistently elevated UV-B levels, which can be up to 2–3 times higher than those found in lowlands [5,6].

The high energy of UV-B radiation causes severe biological damage, including DNA mutations, protein denaturation, and oxidative stress. For aquatic ecosystems, UV-B can penetrate clear water bodies unimpeded, threatening the survival and reproduction of various species [7]. In response, high-altitude organisms have evolved diverse defense mechanisms: for instance, *Euglena* in Dianchi Lake synthesize excessive mycosporine-like amino acids (MAAs) [8]; green algae effectively repair UV-induced DNA damage through photo repair enzymes to maintain genomic stability [9]; Qinghai–Tibet Plateau loaches possess thicker and denser scales, with the development of external protective structures reducing UV-B penetration into deep tissues [10]; plateau cladocerans precisely avoid UV-B radiation through behavioral patterns of ascending only at dawn and dusk [11]. These defense mechanisms involve morphological changes, behavioral modifications, and multi-level physiological adaptations, shaping survival and ecological interactions through alterations in growth, reproductive success, and overall fitness, thereby influencing ecosystem dynamics [12]. The defense strategies of these organisms provide a reference direction for analyzing the UV-B resistance mechanism of *Artemia*.

*Artemia*, known as brine shrimp, can be found at altitudes ranging from −154 m to 5040 m [13]. These extremophiles are able to thrive in hypersaline lakes around the world, showing resilience to various stressors, such as intense UV-B radiation, temperature fluctuations, and osmotic stress [14]. Broad environmental adaptability renders them a valuable model for investigating stress tolerance [15]. Critically, *Artemia* exhibit two distinct reproductive modes—bisexual reproduction and obligate parthenogenesis—offering a unique avenue to dissect how reproductive modes interact with environmental stress tolerance [16]. Research has shown that bisexual individuals outperform parthenogenetic lineages under temperature stress [17,18]; it is unclear if this extends to UV-B resistance. A systematic investigation of *Artemia*’s UV-B resistance at various altitudes is lacking. The influence of reproductive modes on *Artemia*’s UV-B resistance and their interaction with altitude is not well understood. Previous studies have indicated stage-specific sensitivity, with nauplii being more vulnerable than adults [19], but these studies were limited to specific stages and did not account for different altitudinal species/lineages or reproductive modes.

This highlights the need to address the following critical questions: (1) How does UV-B tolerance vary among *Artemia* species/lineages across altitudinal gradients? (2) Do reproductive modes (bisexual vs. parthenogenetic) influence UV-B resistance? If this is the case, how do they interact with altitude? (3) Are there specific patterns of tolerance at different developmental stages (embryos, nauplii, adults)?

Studying the differences in ultraviolet tolerance among six *Artemia* species/lineages with different altitudes and reproductive modes is the first step to gain an in-depth understanding of the mechanisms involved. Comparing UV-B tolerance across six *Artemia* species/lineage with differing altitudes and reproductive modes, we assessed hatching success for embryos without a protective chorion, as well as relative survival rates for nauplii and adults under varying UV-B levels. It is the first time to systematically integrate three core dimensions—altitudinal gradients, reproductive modes, and developmental stages—to reveal the adaptive patterns of UV-B resistance in *Artemia*, thereby providing a new perspective for the study of biological adaptation mechanisms in extreme environments.

## 2. Materials and Methods

Six *Artemia* species/lineages with distinct reproductive modes and altitudinal habitats were utilized in this study, with detailed information on cysts provided in Table 1. Parthenogenetic lineages consisted of GHL, BRK, and EBL, while bisexual species included LGC, ARB, and SFB. All cysts were stored at −20 °C in dark conditions to maintain viability prior to experiments, thereby minimizing batch-specific variability in hatching performance. Four sites of cysts used in the study were generously provided by Dr. SUN Shichun in Ocean University of China, except BRK and EBL. *Artemia* species/lineages detail information can be found in Zheng et al. [13] and Pang et al. [20].

### 2.1. Construction of UV-B Irradiation Device

To replicate solar ultraviolet radiation, the UV-C lamp in the programmable light incubator (GXZ-380D, Ningbo Jiangnan Instrument Factory, Ningbo, China) was replaced with a UV-B lamp (G8TSE, Dongguan Chuangu Lighting Technology Co., Ltd., Dongguan, China). This lamp emits radiation in the wavelength range of 280–320 nm, with a peak emission at 313 nm. A rotating sample platform (Shengzhou Dingshi Electronic Technology Co., Ltd., Shengzhou, China) was positioned 15 cm directly below the UV-B lamp to ensure uniform exposure of all samples. Prior to each use, the UV-B lamp was activated and allowed to run continuously for 30 min to stabilize its irradiance output [21].

### 2.2. UV-B Irradiation Experiments on Artemia Embryos

To ensure the accuracy of the experiment and control variables, we used decapsulated cysts for the experiment.

#### 2.2.1. Experimental Design and Irradiation Protocol

Based on preliminary trials, UV-B dose gradients were set as 0, 1.6, 3.2, 4.8, and 6.4 kJ·m^−2^ for cyst types. For each species/lineage, morphologically uniform cysts were selected under a stereomicroscope (SZN71, Ningbo Shunning Co., Ltd., Ningbo, China). Each dose group was assigned 120 cysts, with three biological replicates.

Cysts were first placed in a 100-mesh sieve and submerged in a solution containing 3% available chlorine and sodium hypochlorite (239305-25ML, Sigma-Aldrich Trading Co., Ltd., Shanghai, China) at 25 °C for 15 min. Then, 1.0% sodium thiosulfate (1065120250, Sigma-Aldrich Trading Co., Ltd., Shanghai, China) was poured over them for 1 min [22]. After decapsulation, cysts were rinsed thoroughly with distilled water (5 times) to eliminate residual reagents, then transferred to six-well culture plates with hatching solution.

All embryo samples were exposed to UV-B radiation using the device described in Section 2.1. Irradiation time for each dose was calculated using the formula: D = P × t (where D = UV-B dose, P = irradiance, t = exposure time) [23]. Post-irradiation, plates were incubated in a programmable light incubator (GTOP-310B2, Zhejiang Tuopu Co., Ltd., Ningbo, China) at 28 °C with a 24-h light photoperiod [24]. The number of hatched nauplii was recorded after 24 h [25,26].

#### 2.2.2. Effect of UV-B Irradiation on *Artemia* Nauplii

Based on preliminary experiments, the UV-B irradiation dose gradients for *Artemia* nauplii were set at 0, 0.2, 0.4, 0.8, and 1.6 kJ·m^−2^.

The prepared hatching solution was poured into the hatching container until it reached approximately half of its volume. *Artemia* embryos were added to the solution and incubated in a constant-temperature incubator at 28 °C under a 24-h light photoperiod to allow hatching. After hatching was completed and the nauplii emerged, 120 active individuals were randomly selected for each dose group and evenly distributed into six-well culture plates containing fresh culture solution (20 individuals per well). Each well was labeled with the corresponding irradiation dose. The irradiation time required for each dose was calculated using the following formula:

D = P × t, where D represents the UV-B dose (kJ·m^−2^), P denotes the irradiance (W·m^−2^), and t is the exposure time (s).

The plates were then placed in the UV-B irradiation device and exposed to the designated doses. Following irradiation, the plates were transferred to a programmable light incubator maintained at 28 °C with a 24 h D (24 h dark) photoperiod to eliminate the influence of photo repair. After 24 h, the number of surviving nauplii in each treatment group was recorded. Each experimental condition was replicated three times.

#### 2.2.3. Effect of UV-B Irradiation on Adult *Artemia*

Based on preliminary experiments, the UV-B irradiation dose gradients for adult *Artemia* were set at 0, 0.8, 1.6, 2.4, and 3.2 kJ·m^−2^.

After the *Artemia* reached sexual maturity—indicated by the appearance of an egg sac in parthenogenetic individuals and the presence of tail-clasping behavior in bisexual individuals—30 adults were selected for each dose group and placed into six-well culture plates containing fresh culture solution. Each well was clearly labeled with the corresponding irradiation dose. The irradiation time required for each treatment was calculated using the formula: D = P × t, where D represents the UV-B dose (J·m^−2^), P is the irradiance (W·m^−2^), and t is the exposure time (s).

Following this period, the culture plates were exposed to UV-B using the irradiation setup. After exposure, the plates were transferred to a programmable incubator set at 28 °C, under continuous darkness (24 h D) to prevent photo repair. The number of surviving adults was recorded at 24, 48, 72, 96, and 120 h post-irradiation. During the observation period, the *Artemia* were fed daily, and distilled water was added regularly to maintain culture conditions.

### 2.3. Calculation of Relative Change

To determine the hatching rate, the number of hatched nauplii was counted under a microscope, and the percentage was calculated using the formula. However, in this study, there were differences in the initial hatching rates of embryos and survival rates of nauplii among the tested groups at the 0 kJ·m^−2^ UV-B dose (hereinafter referred to as the “0-dose”). Therefore, the relative change rate was used for description in this study, with the hatching rate and survival rate at the 0-dose set as the control group: Relative hatching rate (%) = [(Number of hatched individuals under different UV-B doses − Number of hatched individuals under 0 kJ·m^−2^ UV-B dose)/Number of hatched individuals under 0 kJ·m^−2^ UV-B dose] × 100%. The calculation method of the relative survival rate is consistent with that of the relative hatching rate [27]. The effect of UV-B on adults was calculated using the survival rate: Survival rate (%) = (Number of surviving adults/Total number of adults) × 100% [28].

### 2.4. Data Analysis and Processing

All experimental data were statistically analyzed using IBM SPSS Statistics 18.0. One-way and two-way analysis of variance (one-way ANOVA/two-way ANOVA) were performed on the 24-h relative hatching rates of *Artemia* embryos, the 24-h relative survival rates of nauplii exposed to different UV-B doses, as well as the dynamic effects of different UV-B doses on the survival of adult *Artemia* at 24 h, 48 h, 72 h, 96 h, and 120 h and their associations with altitude and reproductive modes. Post hoc significance tests were conducted to assess differences among treatment groups.

## 3. Results

### 3.1. Effects of UV-B Irradiation on Artemia Embryos

As shown in Table 2, altitude, UV-B dose, and reproductive mode all have significant effects on the hatching rate of *Artemia* embryos. The interaction between altitude and reproductive mode, as well as the interaction between reproductive mode and UV-B dose, approached a significant level, while the interaction between altitude and UV-B dose had no significant effect.

**Figure 1:**
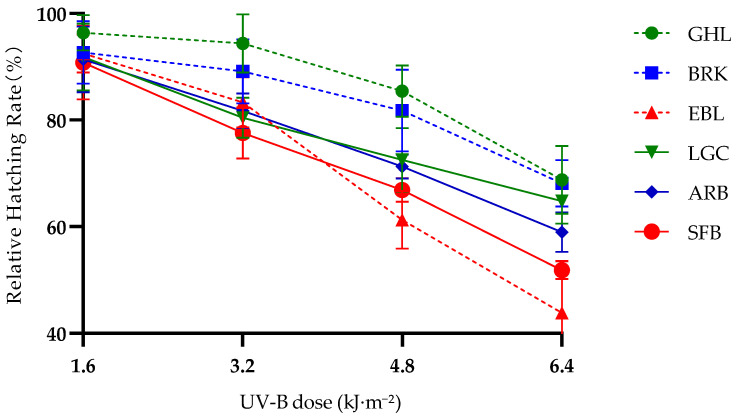
Changes in Relative Hatching Rate of *Artemia* Embryos Under Gradient UV-B Stress at Different Altitudes and Reproductive Modes. Note: Error bars represent the standard deviation, with *n* = 3. Red = bisexual reproduction, Blue = parthenogenesis; Circles = high altitude, Squares = medium altitude, Triangles = low altitude. The relative hatching rate was calculated based on the 0 UV-B control group for each *Artemia* embryo population. The group treated with 0 kJ·m^−2^ was set as the control, and the relative hatching rates of each dose group were calculated based on this control.

As can be seen from Figure 1, regardless of differences in altitude and reproductive mode, the relative hatching rates of embryos from all six *Artemia* species/lineages showed a continuous decline as the UV-B dose increased from 0 to 6.4 kJ·m^−2^.The relative hatching rates of high-altitude species/lineages (LGC, GHL) were consistently higher than those of low-altitude species/lineages (EBL, SFB) and mid-altitude species/lineages (BRK, ARB), and this altitudinal difference became more pronounced with increasing UV-B dose.

Under the same reproductive mode, the relative hatching rates of high-altitude parthenogenetic lineages were higher than those of low-altitude parthenogenetic lineages at all doses. Among bisexual species, the relative hatching rate of LGC (the highest-altitude species) was significantly higher than that of ARB and SFB (mid-to-low-altitude species/lineages); particularly in the high-dose range of 4.8–6.4 kJ·m^−2^, LGC showed the most prominent advantage in relative hatching rate.

Within similar altitude ranges, parthenogenetic lineages exhibited better relative hatching rate performance than bisexual species. In the high-altitude range, the relative hatching rate of the parthenogenetic GHL was higher than that of the bisexual LGC, and the retention ratio of GHL’s relative hatching rate was higher, especially at the dose of 6.4 kJ·m^−2^. In the mid-altitude range, the relative hatching rate of the parthenogenetic BRK was higher than that of the bisexual ARB, with more obvious differences observed at doses of 3.2–4.8 kJ·m^−2^. However, in the low-altitude range, the relative hatching rate of the bisexual SFB was higher than that of the parthenogenetic EBL, but the difference was small.

### 3.2. Effect of UV-B Irradiation on the Survival Rate of Artemia Nauplii

As shown in Table 3, UV-B dose, altitude, and reproductive mode all had extremely significant effects on the survival rate, while the interaction effects between altitude and reproductive mode, between reproductive mode and UV-B dose, and between altitude and UV-B dose were not significant.

**Figure 2:**
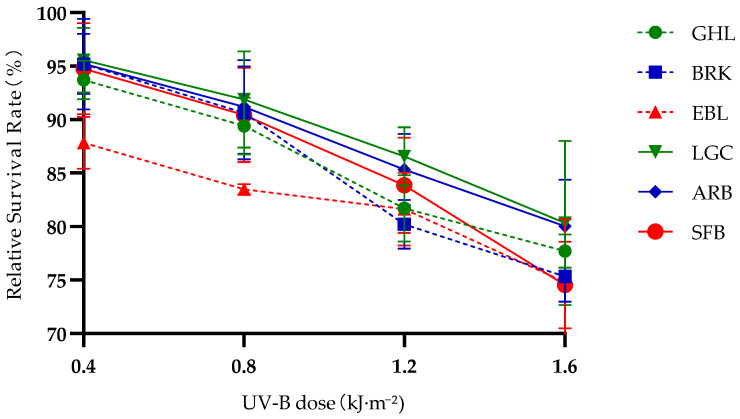
Changes in Relative Survival Rate of *Artemia* Nauplius Under Gradient UV-B Stress at Different Altitudes and Reproductive Modes.

As shown in Figure 2, regardless of differences in altitude and reproductive mode, the relative survival rates of nauplii from all six *Artemia* species/lineages showed a continuous decline as the UV-B dose increased from 0 to 1.6 kJ·m^−2^. At each UV-B dose level, the relative survival rates of *Artemia* nauplii from high-altitude species/lineages were consistently higher than those from mid-altitude and low-altitude species/lineages, with the difference becoming more pronounced as altitude increased.

In terms of differences in reproductive mode, there were regular variations among species/lineages within similar altitude ranges: the relative survival rates of bisexual *Artemia* nauplii were higher than those of parthenogenetic *Artemia* nauplii in each altitude interval. The difference in relative survival rates among *Artemia* species/lineages at low altitudes was relatively large, while the difference among those at high altitudes was relatively small.

### 3.3. Dynamic Effects of UV-B Radiation on the Survival of Adult Artemia and Their Associations with Altitude and Reproductive Mode

**Figure 3:**
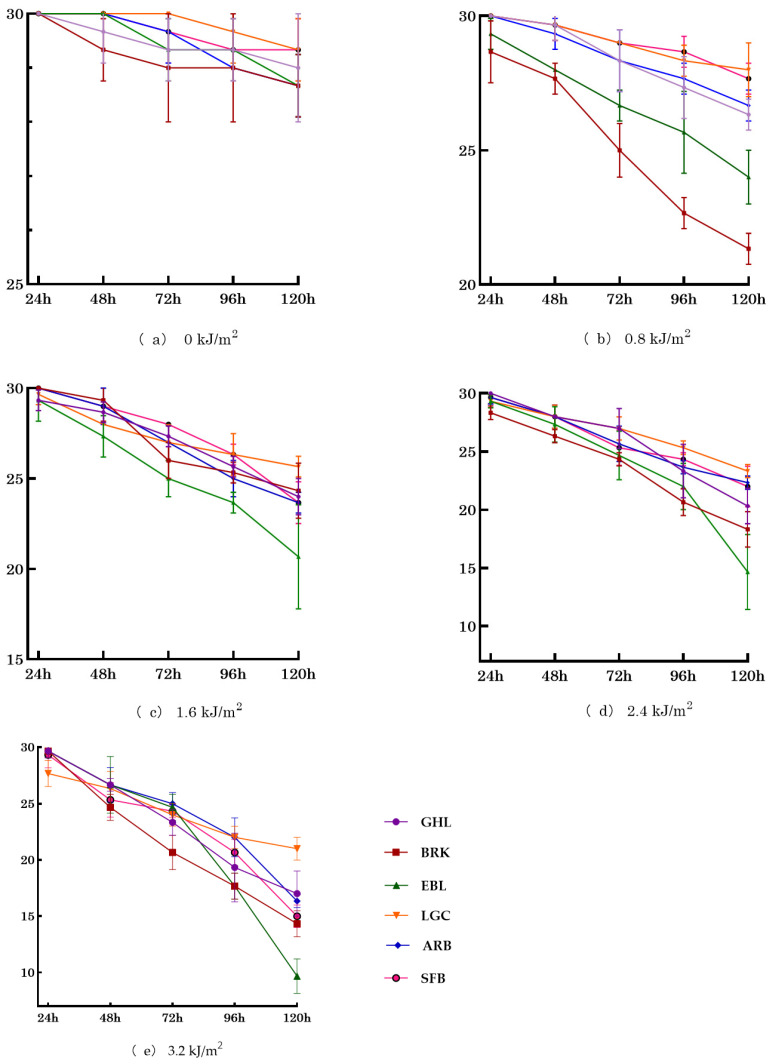
Effect of UV-B Irradiation on Adult *Artemia*. Note: The ordinate in the figure represents the number of surviving *Artemia* (individuals), and the abscissa represents the time after irradiation (h).

As shown in Figure 3a, under 0 kJ·m^−2^ UV-B irradiation, there was no significant change over 120 h (*p* > 0.05). Under 0.8 kJ·m^−2^ irradiation (Figure 3b), minor mortality was observed in LGC, GHL, ARB, and SFB; in contrast, EBL and BRK exhibited higher mortality rates, with the divergence between lineages becoming more pronounced over time. Figure 3c illustrates that exposure to 1.6 kJ·m^−2^ resulted in decreased survival across all six species/lineages, with a decline trend comparable to that seen at 0.8 kJ·m^−2^. At 2.4 kJ·m^−2^ (Figure 3d), survival significantly decreased relative to 1.6 kJ·m^−2^, with lineages from EBL and SFB experiencing the most pronounced reductions. Under 3.2 kJ·m^−2^ irradiation (Figure 3e), survival sharply declined in all species/lineages except those from LGC, where mortality was comparatively lower; notably, nearly all EBL perished. Final survival was highest in the LGC species, followed sequentially by GHL, ARB, SFB, and BRK, with EBL exhibiting the lowest survival.

The number of surviving adults was recorded at 24, 48, 72, 96, and 120 h post-irradiation. Table A1 displays the variations in 24-h survival rates among adult individuals from six *Artemia* species/lineages exposed to different UV-B doses. Significant differences in survival rates are observed at doses of 0.8, 1.6, and 2.4 kJ·m^−2^ (*p* < 0.05), with a nearly significant difference at 3.2 kJ·m^−2^ (*p* = 0.062). The correlation with altitude is weak (−0.279 to 0.204) and not significant at any dose, indicating that altitude-related adaptive mechanisms are not yet prominent within 24 h. The correlation with reproductive mode is significant only at 0.8 kJ·m^−2^ (correlation coefficient 0.5 *).

Table A2 shows that after 48 h of UV-B exposure, significant differences among species/lineages were only observed at the dose of 0.8 kJ·m^−2^ (*p* < 0.05) in terms of the survival rate of adult *Artemia* after 48 h of UV-B exposure. No significant differences were found at other doses. The correlation with altitude was consistently low (0.071–0.361) and not significant, indicating that altitude-related mechanisms were not the dominant factor for short-term survival. The correlation with reproductive mode was significant only at 0.8 kJ·m^−2^ (correlation coefficient 0.589 *), showing that bisexual species had a survival advantage under low doses, possibly due to more efficient damage repair capabilities. This advantage was not present under medium and high doses due to cumulative damage.

Table A3 shows that after 72 h of UV-B exposure, significant differences among species/lineages were observed at doses of 0.8, 1.6, and 3.2 kJ·m^−2^ (*p* < 0.05), with a trend towards significance at 2.4 kJ·m^−2^ (*p* = 0.19). Correlation with altitude was significant at 2.4 kJ·m^−2^ (correlation coefficient 0.511 *), indicating that high-altitude species/lineages may activate specific adaptive mechanisms under medium doses. The correlation with reproductive mode was extremely significant at 0.8 kJ·m^−2^ (0.663 **) and significant at 1.6 kJ·m^−2^ (0.512 *), suggesting that bisexual species have a survival advantage under medium and low doses, possibly due to better long-term repair ability. This advantage diminishes under high doses of stress.

Table A4 shows the survival rate results of adult *Artemia* after 96 h of UV-B exposure. Significant differences were observed among species/lineages at all doses (0.8, 1.6, 2.4, and 3.2 kJ·m^−2^) (*p* < 0.05), indicating consistent differences in tolerance capabilities among species/lineages regardless of dose level. The correlation with altitude was consistently low (0.125–0.268) and not significant, suggesting that altitude has a weak direct impact on adult survival even after long-term exposure. However, the correlation with reproductive mode was highly significant at 2.4 kJ·m^−2^ (0.643 **) and 3.2 kJ·m^−2^ (0.730 **), with the correlation becoming stronger with higher doses. This suggests that bisexual species, with their higher genetic diversity, are better able to adapt to chronic high-intensity damage and exhibit significantly improved survival rates under long-term stress.

Table A5 shows that after 120 h of UV-B exposure, significant differences in the 120-h survival rates were observed among the six *Artemia* species/lineages under all UV-B dose treatments, except for the 0 kJ·m^−2^ irradiation treatment. There was no significant correlation between the altitude of the *Artemia* species/lineages and their 120-h survival rates following irradiation at 0, 0.8, and 1.6 kJ·m^−2^. However, at 2.4 and 3.2 kJ·m^−2^ irradiation levels, a significant positive correlation was detected between altitude and survival rates. The reproductive mode of *Artemia* showed no significant correlation with survival at 0 and 0.8 kJ·m^−2^; in contrast, significant correlations between reproductive mode and 120-h survival were observed at 1.6, 2.4, and 3.2 kJ·m^−2^ irradiation doses.

## 4. Discussion

The present study systematically investigated the effects of UV-B, altitudinal gradients, and reproductive modes on the hatching and survival performance of six *Artemia* species/lineages across three developmental stages. By integrating these three core dimensions for the first time, we revealed three distinct adaptive patterns of *Artemia* in response to UV-B stress, which not only enriches our understanding of the stress adaptation mechanisms of extremophiles but also provides empirical support for the synergistic regulation of environmental and biological traits in shaping species tolerance.

Our results confirmed that high-altitude *Artemia* species/lineages exhibit significantly superior UV-B tolerance compared to low-altitude counterparts, reflected in higher relative hatching rates of embryos and longer survival periods of adults under high-dose UV-B stress. Notably, this altitude-associated tolerance shows distinct developmental stage specificity: the differences between high-altitude and low-altitude strains of *Artemia* mainly manifest under prolonged or stronger UV-B irradiation. Studies have shown that low doses of UV-B can exert positive effects on organisms, promoting the expression of relevant genes and the production of more proteases [29]. However, at high doses, UV-B damages cellular structures and molecular mechanisms [30]. Organisms in high-altitude areas develop greater resistance when exposed to high doses of UV-B. For instance, benthic macroinvertebrates in high-altitude areas develop greater pigmentation to mitigate the impacts of UV-B [31]. Compared with organisms inhabiting low-altitude regions, those in high-altitude areas synthesize a greater abundance of UV-B resistance-related metabolites to safeguard cellular structures [32]. This “high-altitude–high tolerance” pattern is not limited to UV-B resistance; high-altitude *Artemia* species/lineages also show enhanced adaptability to other concurrent extreme environmental factors, such as low temperatures and high salinity in plateau habitats [33]. This “high-altitude–high-tolerance” correlation is not unique to *Artemia*; it is equally prevalent in other biological taxa. For example, reptiles, amphibians, fish, and plants at high altitudes show higher resistance than their counterparts at low altitudes [34,35,36,37,38]. Such comprehensive stress tolerance may stem from long-term directional selection in high-altitude environments, which may drive the evolution of intrinsic protective traits—for example, the formation of thicker cyst shells, or the enhancement of DNA repair enzyme activity and antioxidant capacity [39,40].

Reproductive mode plays a key regulatory role in *Artemia*’s UV-B tolerance, but this effect varies significantly across developmental stages, presenting a distinct stage-specific pattern. At the embryonic stage, parthenogenetic lineages showed higher relative hatching rates than bisexual species at the same altitude and UV-B dose, contradicting their lower tolerance at the naupliar and adult stages. This unique phenomenon may be related to the maternal environment: parthenogenetic *Artemia* in the wild may ingest more food sources rich in MAAs and transfer these compounds to their embryos through reproductive organs, thereby enhancing embryonic resistance to UV-B damage [41,42,43]. In contrast, the naupliar and adult experiments were conducted under standardized laboratory feeding conditions, eliminating dietary interference and thus reflecting the intrinsic differences between reproductive modes. Because parthenogenetic females may transfer MAAs or other protective compounds to embryos, some of the observed UV-B tolerance may reflect maternal effects rather than intrinsic genetic differences. In the future, raising synchronized generations under uniform laboratory conditions will help separate maternal influence from genetic factors.

At the naupliar stage, bisexual species maintained higher relative survival rates than parthenogenetic lineages across all altitude ranges. At the adult stage, bisexual species showed more stable survival under prolonged UV-B exposure (96–120 h), with significant positive correlations between reproductive mode and survival rate (r = 0.533 *–0.74 **). The phenomenon that bisexual reproduction confers higher UV-B resistance than parthenogenesis also exists in other taxa. For example, bisexual *Eucypris virens* exhibit higher UV-B resistance than parthenogenetic individuals [44]. In addition to UV-B resistance, bisexual species also show better tolerance to climatic stress, cold stress, and a variety of other environmental stresses. This phenomenon has been reported in European blueberry psyllid, aphids, and plants [45,46,47]. Under the same altitude conditions and UV-B stress, the correlation of “bisexual reproduction–high resistance” is remarkably evident in *Artemia* at both the adult and naupliar stages. SFB has become an invasive species in the Mediterranean region, and, compared with the native parthenogenetic *Artemia* lineages in the Mediterranean Sea, it has a wider tolerance range to extreme environments. Toxicity tests conducted on it show that the enzymes in SFB exhibit stage-specific resistance and possess a gene-regulated stress-resistant molecular chaperone system [48]. Studies have shown that the bisexual *Artemia* from Lake Urmia inherently exhibit stronger tolerance to UV-B and salinity, and this adaptability may also be associated with their inherent stress-resistant genes [49]. However, taxa that exhibit both bisexual and parthenogenetic reproductive modes are relatively scarce. This necessitates additional data to validate the observed patterns, refine the existing framework, and enhance the robustness of this conclusion [50].

In addition to the above two adaptive patterns, *Artemia* possesses a third adaptive pattern. Our study clearly demonstrated that *Artemia*’s UV-B tolerance varies significantly with developmental stages, following the pattern of “adults > nauplii”. Adults showed the strongest resistance: they could maintain stable survival under moderate UV-B doses (0.8–2.4 kJ·m^−2^) in the short term (24–48 h) and tolerate higher doses for longer periods. This advantage is likely due to their mature physiological systems, sufficient energy reserves, and complex cuticle composed of chitin, proteins, and minerals—all of which provide structural protection and support efficient damage repair [51,52]. The high tolerance of adult *Artemia* to UV-B may also be associated with other protective mechanisms, such as pigment deposition, regulation via endogenous enzymatic/non-enzymatic systems, or light-avoidance behavior. Notably, similar protective mechanisms have been documented in both Antarctic hair grass and bdelloid rotifers [53,54]. Although these indicators were not directly measured in this study, these findings still provide a direction for subsequent exploration of the specificity of protective mechanisms in adult individuals. Even under low UV-B doses, the relative survival rate of nauplii decreases significantly. This may be because nauplii exhibit immature physiological functions and limited energy reserves, which hinder their ability to cope with the dual challenges of UV-B-induced energy consumption and the metabolic demands of damage repair [55]. This “adult developmental stage–high resistance” association is also not exclusive to *Artemia*. Fish are a typical representative of aquatic animals. Adult fish can develop a thicker epidermal layer and scales to resist UV-B radiation [56].

## 5. Conclusions

By analyzing the UV-B tolerance of six *Artemia* species/lineages across different altitudes (113–4700 m), reproductive modes (bisexual reproduction and parthenogenesis), and developmental stages (embryos, nauplii, and adults), this study revealed the core adaptive characteristics of *Artemia* in response to UV-B stress. *Artemia* at high altitudes have developed an “altitude adaptive barrier” through long-term selection under extreme UV-B radiation, exhibiting significantly higher UV-B resistance than low-altitude populations. Particularly under high-dose UV-B exposure, the declines in their embryo relative hatching rates and adult survival rates are much smaller. At the same altitude, adult *Artemia* with bisexual reproduction show far greater resistance to long-term high-intensity UV-B than parthenogenetic lineages. However, parthenogenetic *Artemia* have a higher embryo relative hatching rate, presenting developmental stage-specific differences. Additionally, the UV-B resistance of adult *Artemia* is significantly higher than that of nauplii: low-dose UV-B can already affect nauplii survival, while adults can tolerate medium-dose UV-B. Among these findings, high-altitude bisexual *Artemia* are preferred materials for UV-B-resistant breeding. Meanwhile, this study clarifies the synergistic regulatory mechanism between altitude and biological traits in shaping *Artemia*’s UV-B tolerance, providing theoretical support for research on biological adaptation to extreme environments and stress-resistant breeding practices.

## Figures and Tables

**Table 1:** Geographical and reproductive mode of *Artemia* species and lineages.

Source of *Artemia*	Reproductive Mode	Longitude	Latitude	Altitude	Abbreviations
Gahai Lake, Qinghai	Parthenogenesis	97°33′37″ E	37°8′37″ N	2851 m	GHL
Barkol Salt Lake, Xinjiang	Parthenogenesis	92°44′42″ E	43°40′51″ N	1582 m	BRK
Ebinur Lake, Xinjiang	Parthenogenesis	82°43′43″ E	44°52′15″ N	252 m	EBL
Laguo Co, Tibet (*A. tibetiana*)	Bisexual	82°27′38″ E	32°31′30″ N	4700 m	LGC
Alxa Right Banner, Inner Mongolia (*A. sinica*)	Bisexual	101°41′9″ E	39°12′58″ N	1339 m	ARB
San Francisco Bay, USA (*A. franciscana*)	Bisexual	122°25′2″ W	37°46′8″ N	113 m	SFB

Note: *Artemia* abbreviations are consistently used throughout the text.

**Table 2:** The significance of various influencing factors on the hatching rate of *Artemia* embryos.

Influencing Factors	*p*
Altitude	0.002 *
UV-B	<0.01 **
Reproductive mode	0.022 *
Altitude * Reproductive mode	0.07
Reproductive mode * UV-B	0.069
Altitude * UV-B	0.269

Note: * *p* < 0.05, ** *p* < 0.01.

**Table 3:** The significance of various influencing factors on the survival rate of *Artemia* nauplii.

Influencing Factors	*p*
Altitude	0.041 *
UV-B	<0.01 **
Reproductive mode	0.009 **
Altitude * Reproductive mode	0.499
Reproductive mode * UV-B	0.692
Altitude * UV-B	0.815

Note: * *p* < 0.05, ** *p* < 0.01.

## Data Availability

The raw data supporting the conclusions of this article will be made available by the authors on request.

## Appendix A

**Table A1:** Significance of differences in 24-h survival among adult individuals of six *Artemia* species/lineages under varying UV-B doses and their correlations with altitude and reproductive mode.

UV-B Dose	*p*	Correlation Coefficient with Altitude	Correlation Coefficient with Reproductive Mode
0.8 kJ·m^−2^	<0.05	0.204	0.5 *
1.6 kJ·m^−2^	<0.05	−0.122	0.41
2.4 kJ·m^−2^	<0.05	0.1	0.246
3.2 kJ·m^−2^	0.062	−0.279	−0.444

Note: *p* < 0.05 indicates a significant difference at the 0.05 level; * indicates a significant correlation at the 0.05 level.

**Table A2:** Significance of differences in 48-h survival among adult individuals of six *Artemia* species/lineages under varying UV-B doses and their correlations with altitude and reproductive mode.

UV-B Dose	*p*	Correlation Coefficient with Altitude	Correlation Coefficient with Reproductive Mode
0 kJ·m^−2^	0.136	−0.183	0.447
0.8 kJ·m^−2^	<0.05	0.361	0.589 *
1.6 kJ·m^−2^	0.087	0.071	0.116
2.4 kJ·m^−2^	0.19	0.143	0.409
3.2 kJ·m^−2^	0.526	0.135	0.032

Note: *p* < 0.05 indicates a significant difference; * indicates a significant correlation at the 0.05 level.

**Table A3:** Significance of differences in 72-h survival among adult individuals of six *Artemia* species/lineages under varying UV-B doses and their correlations with altitude and reproductive mode.

UV-B Dose	*p*	Correlation Coefficient with Altitude	Correlation Coefficient with Reproductive Mode
0 kJ·m^−2^	0.488	0.113	0.462
0.8 kJ·m^−2^	<0.05	0.214	0.663 **
1.6 kJ·m^−2^	<0.05	0.228	0.512 *
2.4 kJ·m^−2^	0.2	0.511 *	0.209
3.2 kJ·m^−2^	<0.05	−0.2	0.458

Note: *p* < 0.05 indicates a significant difference; * indicates a significant correlation at the 0.05 level; ** indicates a significant correlation at the 0.01 level.

**Table A4:** Significance of differences in 96-h survival among adult individuals of six *Artemia* species/lineages under varying UV-B doses and their correlations with altitude and reproductive mode.

UV-B Dose	*p*	Correlation Coefficient with Altitude	Correlation Coefficient with Reproductive Mode
0 kJ·m^−2^	0.781	0.122	0.1
0.8 kJ·m^−2^	<0.05	0.125	0.688 **
1.6 kJ·m^−2^	<0.05	0.367	0.449
2.4 kJ·m^−2^	<0.05	0.25	0.643 **
3.2 kJ·m^−2^	<0.05	0.268	0.730 **

Note: *p* < 0.05 indicates a significant difference; ** indicates a significant correlation at the 0.01 level.

**Table A5:** Significance of differences in 120-h survival among adult individuals of six *Artemia* species/lineages under varying UV-B doses and their correlations with altitude and reproductive mode.

UV-B Dose	*p*	Correlation Coefficient with Altitude	Correlation Coefficient with Reproductive Mode
0 kJ·m^−2^	0.618	0.11	0.268
0.8 kJ·m^−2^	<0.05	0.226	0.343
1.6 kJ·m^−2^	<0.05	0.437	0.74 **
2.4 kJ·m^−2^	<0.05	0.560 *	0.73 **
3.2 kJ·m^−2^	<0.05	0.767 **	0.533 *

Note: *p* < 0.05 indicates a significant difference; * indicates a significant correlation at the 0.05 level; ** indicates a significant correlation at the 0.01 level.
